# The Companion Pandemic to COVID-19: The Use of Informal Practices to Access Public Healthcare Services in the European Union

**DOI:** 10.3389/ijph.2022.1604405

**Published:** 2022-10-19

**Authors:** Adrian V. Horodnic, Colin C. Williams, Răzvan Ionuț Drugă

**Affiliations:** ^1^ Faculty of Medicine, Grigore T. Popa University of Medicine and Pharmacy, Iași, Romania; ^2^ Management School, The University of Sheffield, Sheffield, United Kingdom; ^3^ Faculty of Economics and Business Administration, Alexandru Ioan Cuza University of Iași, Iași, Romania

**Keywords:** COVID-19, informal payments, personal connections, healthcare access, asymmetry formal-informal institutions, trust in authorities

## Abstract

**Objectives:** The objective of this paper is to evaluate the use of informal payments and personal connections to gain preferential access to public health services during the COVID-19 pandemic and to propose effective policy measures for tackling this phenomenon.

**Methods:** Using data from 25,744 patients in the European Union, six different scenarios are analyzed in relation to making informal payments and/or relying on personal connections to access public healthcare services. To evaluate the propensity to engage in informal practices in healthcare, probit regressions with sample selection and predicted probabilities are used. Robustness checks are also performed to test the reliability of the findings.

**Results:** For each scenario, a statistically significant association is revealed between the propensity to make informal payments and/or rely on personal connections and the asymmetry between the formal rules and the patients’ personal norms and trust in public authorities.

**Conclusion:** To tackle informal practices in healthcare, policy measures are required to reduce the asymmetry between the formal rules and personal norms by raising trust in public authorities.

## Introduction

The COVID-19 pandemic, together with the responses adopted by public authorities, have generated different types of behaviors. Unfortunately, one such behaviour has been engagement in corrupt actions [1]. One such corrupt informal practice used during this period has been informal payments, sometimes referred to as “under the table” payments [2], “out-of-pocket payments” [3] and “unofficial” payments [4]. Along with gifts, they differ from declared official payments in that they are not mediated by the state or brought to their attention [5]. Informal payments in the health sector are here defined as direct contributions made in cash or in kind by patients or others acting on their behalf, to health care providers for services that the patients are entitled to [6]. According to the literature, there are three main reasons why informal payments are used. These are: “excessive red tape,” “to solve a problem” and “to do something that goes against legal codes” [5, p. 386]. They help solve access to health services.

Another corrupt informal practice has been for patients to use personal connections during the COVID-19 pandemic either to gain preferential access to services, benefit from a service superior to the classic ones [1] or to gain faster access to services, jumping the waiting list. Various terms are adopted in different countries to describe the system of using personal connections, including “dear brothers” in Finland and “ties” in the Balkans [7]. According to Transparency International, based on data collected in 2020, over the last year, 29% of instances when personal connections have been used relate to gaining preferential access to public clinics and hospitals [1].

Comparing these two types of informal practice, patients using personal connections to receive preferential access to health services is more easily accepted by the public [8]. Sometimes, moreover, the two practices are directly related in that a lack of personal connections can result in difficulties in making informal payments [9]. But both practices however create losers and winners among patients, affecting the allocation of public healthcare resources, reducing healthcare access and raising social equity issues [10]. Offering (preferential) treatment based on informal practices is made at the expense of medical services needed by other patients, often marginalised socio-economic groups [8,12–16]. When patients consider that the norm is to offer an informal payment, not having the financial resources to offer that payment may lead to the decision to postpone the visit to the healthcare professional, visit less specialized medical staff or even give-up healthcare services [11,17]. The situation is even worse when demand exceeds the available supply for healthcare services or when are service disruptions (like happened during the COVID-19 pandemic [13,19]. Therefore, due to their impact on health systems and patients, these informal practices are a core issue on the policy agendas of various international organizations, such as OECD [19], European Commission [20], World Health Organization [21] or Transparency International [11].

In the European Union during the COVID-19 pandemic, informal payments have been found to be more common in some countries more than others, and most common in Romania (where 22% of patients have used informal payments) and Bulgaria (19%), while the use of personal connections to gain preferential access to health services has been most common in Czechia (where 54% of patients have used personal connections), Hungary (41%) and Portugal (46%) [1]. Given that formal and informal practices are inextricably inter-linked [22], the high use of informal practices during the pandemic suggest that unless action is taken to stem their growth, in some countries the situation might be reached where informal practices will become the norm rather than an exception [23], and ever more patients will engage in such practices to gain access to health services [24].

To discourage informal practices, one method could be to deter the engagement of health services staff in such practices, using harsher penalties or sanctions (e.g., “naming and shaming”) [8]. Given the lack of effectiveness of this policy approach, an alternative policy approach has emerged in recent years grounded in institutional theory. This argues that all societies have both formal institutions (laws and regulations) and informal institutions (citizens norms and values). When there is asymmetry between these formal and informal institutions, informal payments emerge [25,26]. Indeed, in the field of informal practices, the role of socio-cultural factors such as the values at societal level has been previously documented in many studies [27–29]. According to these studies the informal payments are rooted in culture and evolved throughout the historical context [28]. Differences also exist between informal practices used in rural and urban areas. While in the rural space informal payments to the medical staff usually takes the form of provision of gifts, in the urban setting the phenomenon is more complex as it requires at least a loose connection (e.g., a friend of a friend) to put the patient in touch with the medical staff in order to be able to make an informal payment, which is mostly monetary [29,30]. Similarly, cultural differences between countries are identified in the literature of informality. For example, in post-communist societies informal practices are found as being more acceptable by the citizens [14,31]. In this context therefore, the policy approach is to reduce the asymmetry between the formal rules and the personal norms of staff and patients regarding the acceptability of informal practices [25,26]. This asymmetry mostly arises when there is a culture of gratitude (a custom of showing appreciation; informal practices being considered “legitimate” in the viewpoint of informal institutions) embedded in society [13] or when there is a lack of trust by citizens in the authorities [32]. The solution, therefore, is to bolster trust in public authorities [33], not least by providing high quality services and reducing perceptions of corruption in public service provision [34]. Indeed, the lack of trust in receiving high quality medical services in the event of not offering informal payments, based on rumours about inadequate treatments or on previous experience, represent a strong enough argument for a patient to make such informal payments [29,35]. For instance, it is plausible to assume that one can find acceptable and use informal practices to secure access to healthcare services for his child or parent in need of care that is not available (e.g., not meeting strict selective criteria (due to lack of funds) to access treatment). The root of the asymmetry (which makes informal practice acceptable) is the failure or imperfections of the formal institutions (e.g., lack of resources).

Based on this, we here test the following hypotheses related with institutional asymmetry thesis:

H1 : Patients having personal norms and values in asymmetry with formal rules are more likely to resort to informal practices to access public healthcare services.

H2 : Patients with low trust in public authorities are more likely to resort to informal practices to access public healthcare services.

## Methods

We here report the results of The Global Corruption Barometer (GCB)—European Union 2021 [1], which involved 40,663 computer assisted telephone interviews conducted in 27 Member States of the European Union (EU-27), of which 25,744 were conducted with patients who had contact with a public clinic or hospital. On behalf of Transparency International, adults aged 18 years and older were interviewed during the Covid-19 pandemic (starting mid-October until December 2020) on issues related with corruption practices. Representative samples by region were achieved, with a minimum of 300 respondents by NUTS 1 level (Eurostat’s Nomenclature of Territorial Units for Statistics) [36,37].

To analyse the above hypotheses and to provide a clear picture of the informal practices in healthcare and the link between these practices and the trust in public authorities and the asymmetry between personal norms and formal rules (i.e., institutional asymmetry), we report six different scenarios. Each scenario was created to capture various mixtures of informal practices. As Table 1 displays, the first scenario (A) includes in the analysis the total number of respondents who used both personal connections and informal payments for accessing public healthcare services. The second scenario (B) includes in the analysis only those who used both these informal practices more often, at least a few times. Scenarios C and D analyse the use of personal connections, firstly looking to all respondents that declared using only their connections to access public healthcare services and secondly, looking only to those who use this practice more often. The same strategy is used in scenarios E and F but in respect with the use of informal payments to access public healthcare services.

**TABLE 1:** Scenarios considered for informal practices in healthcare (Global Corruption Barometer—European Union, Europe, 2021).

Scenarios	Informal practices in healthcare						
	Use of personal connections to access public healthcare services			and	Use of informal payments to access public healthcare services		
	Once or twice	or	Few times/Often		Once or twice	*or*	Few times/Often
Scenario A	Yes		Yes		Yes		Yes
Scenario B	No		Yes		No		Yes
Scenario C	Yes		Yes		No		No
Scenario D	No		Yes		No		No
Scenario E	No		No		Yes		Yes
Scenario F	No		No		No		Yes

The above scenarios were created based on patients’ answers on whether they used (past 12 months prior to survey, during the COVID-19 pandemic) 1) personal connections or 2) informal payments to get assistance or services needed from a public clinic or hospital. Indeed, only respondents who had contact with a public clinic or hospital in the past 12 months answered these questions. However, it can be reasonably assumed that the largest part of the reported informal practices happened during the Covid-19 crisis, considering that the fieldwork to collect data started in mid-October and ended in December 2020 and by the end of January 2020 the World Health Organization classified the coronavirus outbreak as an emergency of international concern and by mid-March 2020 Europe became the core of the epidemic [38].

To analyze H1 regarding the asymmetry between personal norms and the formal rules (i.e., institutional asymmetry), the acceptability of corrupt behavior is used, based on an attitudinal question about how “acceptable it is for the government to engage in corruption as long as it delivers good results.” High acceptability means high asymmetry level. To analyze H2 regarding trust in public authorities, a Trust in Public Authorities Index for each patient is constructed. This is based on patients trust in the national and local government. The index is normalized on a scale from 0 to 1, where 0 means low trust and 1 means high trust in public authorities.

Considering the dichotomous variables created for each scenario and the fact that informal practices are observable only for those respondents who had contact with a public clinic or hospital, we conducted a probit regression with sample selection. To consider the selection issue [39] in the subset of data, a selection equation controlling for age, education, residency area (urban/rural) and region was used. Indeed, previous studies have shown that these variables are associated with healthcare utilisation and they can be used as predictors for the likelihood to had contact with a public clinic or hospital [40]. Socio-demographic control variables are also used to evaluate the propensity to engage in informal practices, akin to other studies evaluating these practices in healthcare [41]. Details about the variables used in the analysis can be consulted in Supplementary Table S1. Predicted probabilities to engage in informal practices are then computed to graphically portray the results and help interpret the findings. Moreover, robustness checks are performed to test the reliability of the findings. Firstly, a probit regression with sample selection using imputed missing data is performed (multivariate imputations; details in Supplementary Table S2) and secondly, due to the hierarchical nature of the data (patients within countries), a multilevel mixed-effects probit regression is conducted to control for country effect.

## Results

Out of 40,663 respondents from the European Union Member States, 25,774 used healthcare services in the past 12 months prior to the survey. As Table 2 displays, there are cross country and cross regional variations in the use of informal practices in public healthcare services. With 35.1% of patients in East-Central Europe, 33.9% in Southern Europe, 27.9% in Western Europe and 18.6% in Nordic countries using at least one informal practice to access public healthcare services, the finding is that this is not some minor practice. Starting with those who used both informal connections and informal payments for accessing public healthcare services (scenario A), the prevalence is higher in East-Central Europe with 9.4% of those using healthcare services making use of both these informal practices, followed by Southern Europe, Western Europe and Nordic nations where only 2.1%, 1.8% and 0.6% respectively of those using healthcare services employed both informal practices.

**TABLE 2:** Personal connections and informal payments by patients for accessing public healthcare services: by country and scenario considered (%; N = 25,774; Global Corruption Barometer—European Union, Europe, 2021).

	N	TIP	Scenario A	Scenario B	Scenario C	Scenario D	Scenario E	Scenario F
			PCH and IPH	PCH and IPH (frequently)	PCH	PCH (frequently)	IPH	IPH (frequently)
East-Central Europe	10,859	35.1	9.4	3.5	22.4	10.4	3.8	1.3
Romania	1937	43.4	14.9	7.8	21.1	9.8	6.7	4.1
Bulgaria	1,585	38.2	13.6	6.0	18.6	9.0	5.5	2.1
Hungary	633	47.7	12.2	5.7	28.8	13.4	6.3	1.5
Lithuania	716	33.0	11.3	5.0	13.8	7.6	7.6	2.7
Croatia	631	39.4	11.3	5.7	24.1	14.0	3.6	0.3
Czechia	615	56.1	8.5	1.9	45.8	19.3	1.8	0.7
Poland	1,534	39.2	7.4	2.7	28.9	15.0	2.4	1.0
Slovakia	1,336	26.4	6.5	1.9	16.5	7.7	2.9	0.7
Latvia	543	34.3	6.1	2.3	23.7	10.4	3.8	2.3
Slovenia	623	19.7	2.8	0.5	15.2	3.6	1.7	0.2
Estonia	706	12.9	1.3	0.5	10.7	4.5	0.7	0.3
Southern Europe	4,885	33.9	2.1	1.4	30.3	17.9	0.5	0.2
Greece	652	32.4	8.3	3.7	21.9	12.2	2.0	0.9
Malta	355	31.3	3.6	0.8	27.2	16.4	0.0	0.0
Cyprus	278	27.1	3.1	1.5	23.9	8.9	0.2	0.0
Italy	1,029	29.0	2.4	1.7	26.1	18.3	0.4	0.1
Portugal	816	43.9	1.5	1.0	41.9	25.7	0.5	0.0
Spain	1755	35.6	1.0	0.5	34.4	21.1	0.2	0.1
Western Europe	7,672	27.9	1.8	0.7	24.9	11.0	0.7	0.1
Belgium	564	33.5	5.4	2.3	26.3	13.7	1.8	0.2
Austria	463	37.9	4.8	1.4	31.4	11.7	1.2	0.0
France	2,115	37.4	1.7	0.5	34.9	18.0	0.8	0.2
Germany	2,784	18.2	1.3	0.2	15.8	6.2	0.6	0.1
Luxembourg	359	28.0	0.9	0.4	27.1	10.0	0.0	0.0
Ireland	619	24.0	0.7	0.2	22.1	8.1	0.8	0.1
Netherlands	768	18.1	0.6	0.2	17.5	9.7	0.0	0.0
Nordic Nations	2,358	18.6	0.6	0.3	17.9	7.9	0.0	0.0
Denmark	752	19.3	0.8	0.5	18.5	7.5	0.0	0.0
Finland	816	20.7	0.5	0.3	20.0	9.0	0.0	0.0
Sweden	790	16.1	0.5	0.0	15.6	7.3	0.0	0.0

Notes: N, number of patients surveyed; TIP, total informal practices (personal connections or informal payments); PCH, personal connections in healthcare; IPH, informal payments in healthcare; figures computed with survey weighting scheme; scenario estimates may not add to total informal practices (TIP) due to rounding.

Source: author`s calculations based on data from 2^nd^ (2021) Global Corruption Barometer (GCB)—EU [1].

Moving to those who use both types of informal practices to access healthcare services more often (Scenario B), suggesting that the use of informal practices is rather a norm for them and not an exceptional event happening once or twice, the result is that less than a half of respondents declaring using connections and informal payments to access healthcare services do so on a regular basis. The picture is the same as in the first scenario with a higher prevalence in East-Central Europe (3.5%) and lower prevalence in Southern Europe (1.4%), Western Europe (0.7%) and Nordic nations (0.3%). However, this is not the case when analyzing the use of personal connections for accessing public healthcare services. Starting with all those declaring using only their connections for accessing public healthcare services (Scenario C), the finding is that this practice is rather extensive with one in five or more of the healthcare users making use of their informal connections. The practice is more prevalent in Southern Europe (30.3% of patients), Western Europe (24.9%) and East-Central Europe (22.4%) and less prevalent in the Nordic nations where 17.9% of healthcare users declared that they have used personal connections to access public healthcare services. A similar regional ranking is observed when analyzing those who use on a more regular basis the informal connections to access the public healthcare services (Scenario D). Finally, moving to those who used only informal payment to access public healthcare services, the discrepancies between regions and countries are lower. However, the practice is more prevalent in East-Central Europe (3.8% of healthcare users) and not existing at all in the Nordic countries (Scenario E). This is an interesting result and shows that all Nordic nations as well as in some of the countries from Western and Southern Europe (Malta, Luxembourg and the Netherlands) patients do not use solely informal payments. They only make informal payments simultaneously with using personal connections as Scenario A displayed. As such, informal payments in these countries occur only when they trust the person who made the link between the patient and the healthcare provider. A similar picture is found when investigating those who only used informal payments in order to access public healthcare services, but did so on a more regular basis (Scenario F).

Starting to investigate the link between the informal practices used for accessing public healthcare services and the level of asymmetry between the formal and informal institutions as well as the trust in public authorities, Table 3 display the prevalence of these practices by the level of institutional asymmetry and the self-assessed level of trust in public authorities. The finding is that for all the analyzed scenarios, the use of informal practices in healthcare is higher with a high level of institutional asymmetry and a lower level of trust in public authorities.

**TABLE 3:** Personal connections and informal payments by patients for accessing public healthcare services: by formal-informal institutions asymmetry, trust in public authorities and scenario considered (%; N = 25,774; Global Corruption Barometer—European Union, Europe, 2021).

	Scenario A	Scenario B	Scenario C	Scenario D	Scenario E	Scenario F
	PCH and IPH	PCH and IPH (Frequently)	PCH	PCH (Frequently)	IPH	IPH (Frequently)
Institutional Asymmetry Thesis						
Asymmetry formal-informal institutions^a^						
Low asymmetry level	3.8	1.5	23.7	11.8	1.4	0.5
Medium asymmetry level	5.3	2.1	24.3	11.8	2.4	0.8
High asymmetry level	8.4	3.8	28.6	13.3	3.0	1.2
Trust in Public Authorities Index^b^						
Below mean	6.5	2.8	26.8	13.5	2.2	0.8
Above mean	2.2	0.8	21.2	10.0	1.2	0.3

^a^ High asymmetry = personal norms, values not in accordance with formal rules.

^b^ Index score: 0 to 1 (high score = high trust in public authorities).

Notes: PCH, personal connections in healthcare; IPH, informal payments in healthcare;

Source: author`s calculations based on data from 2^nd^ (2021) Global Corruption Barometer (GCB)—EU [1].

Analyzing the results of the descriptive statistics, the finding therefore, is that informal practices exist across all European member states although with a different prevalence, and tend to be more common with higher institutional asymmetry and lower trust in public authorities. To explore whether these findings remain significant when other control variables are included in the analysis, Table 4 displays the results of a probit regression with sample selection. Indeed, the estimates of probit equations for the analysed scenarios would be inconsistent if ignoring the selection into healthcare user status, as the results of the likelihood ratio test of independent equations reveal. As the selection equations shows, there is a positive significant association between the education, age and residency area and healthcare services use. Older individuals, those more educated as well as those living in small- or middle-sized towns are more likely to use healthcare services.

**TABLE 4:** Probit regression (with sample selection) of the propensity to engage in informal practices in healthcare (personal connections and informal payments by patients) in Europe: by scenario (Global Corruption Barometer—European Union, Europe, 2021).

		Model 1	Model 2	Model 3	Model 4
		Scenario A	Scenario B	Scenario C	Scenario D
		PCH and IPH (total)	PCH and IPH (frequently)	PCH (total)	PCH (frequently)
PCH and IPH	*Control Variables*				
	Gender (R: Male)				
	Female	0.065** (0.027)	0.021 (0.037)	0.018 (0.018)	0.047** (0.020)
	Age (exact age)	−0.009*** (0.001)	−0.007*** (0.002)	−0.006*** (0.001)	−0.006*** (0.001)
	Education (R: Primary, Secondary)				
	Tertiary	0.219*** (0.029)	0.163*** (0.040)	−0.020 (0.020)	−0.059** (0.023)
	Occupation (R: Employed)				
	Not working, Homemaker	−0.115** (0.046)	−0.119* (0.063)	0.031 (0.031)	0.058* (0.035)
	Retired	−0.099**(0.047)	−0.103 (0.064)	0.011 (0.029)	0.036 (0.033)
	Student	−0.030 (0.071)	−0.001 (0.098)	0.115** (0.050)	0.064 (0.058)
	Household income (R: Enough to buy what wanted)				
	Enough to buy what is needed	0.130*** (0.032)	0.101** (0.046)	0.067*** (0.020)	0.081*** (0.023)
	Manage with difficulties	0.252*** (0.040)	0.257*** (0.054)	0.110***(0.028)	0.133*** (0.031)
	Not enough to buy what is needed^1)^	0.298***(0.047)	0.300***(0.063)	0.081** (0.035)	0.120*** (0.039)
	Residency area (R: Rural area or village)				
	Small or middle-sized town	0.098*** (0.035)	0.090* (0.047)	0.015 (0.022)	0.031 (0.025)
	Large town	0.115*** (0.035)	0.094** (0.048)	−0.041* (0.023)	-0.029 (0.027)
	*Institutional Asymmetry Thesis*				
	Asymmetry formal-informal institutions (R: Low asymmetry level)				
	Medium asymmetry level	0.170*** (0.047)	0.197*** (0.064)	0.037 (0.035)	0.021 (0.040)
	High asymmetry level	0.351*** (0.032)	0.374*** (0.043)	0.125*** (0.025)	0.104*** (0.028)
	Trust in Public Authorities Index	−0.998*** (0.055)	−1.087*** (0.076)	−0.456*** (0.036)	-0.511*** (0.043)
	Constant	−1.217*** (0.071)	−1.621*** (0.097)	0.022 (0.068)	-0.180** (0.077)
HU	Age (exact age)	0.002*** (0.001)	0.002*** (0.001)	0.001 (0.001)	0.002*** (0.001)
	Education (R: Primary, Secondary)				
	Tertiary	0.208*** (0.015)	0.203*** (0.015)	0.191*** (0.014)	0.193*** (0.015)
	Residency area (R: Rural area or village)				
	Small or middle-sized town	0.036** (0.017)	0.030* (0.018)	0.033** (0.016)	0.035** (0.017)
	Large town	-0.016 (0.018)	−0.023 (0.018)	−0.029* (0.017)	-0.026 (0.017)
	European Region (R: East-Central Europe)				
	Western Europe	0.041** (0.016)	0.060*** (0.016)	0.060*** (0.016)	0.057*** (0.016)
	Southern Europe	0.336*** (0.020)	0.350*** (0.021)	0.406*** (0.019)	0.428*** (0.019)
	Nordic Nations	0.738*** (0.030)	0.762*** (0.031)	0.690*** (0.030)	0.687*** (0.031)
	Constant	-0.152*** (0.027)	−0.218*** (0.028)	0.053** (0.025)	-0.093*** (0.026)
Observations		32,657	31,904	37,786	34,831
Censored		14,642	14,642	14,642	14,642
Uncensored		18,015	17,262	23,144	20,189
χ2		793.2	444.2	470.3	373.4
p>		0.000	0.000	0.000	0.000
LR test of indep. eqns. (rho = 0): p>		0.000	0.000	0.000	0.000

*Notes*: Patients who used a specific informal practice vs. patients who never used that informal practice; Significant at *p < 0.1, **p < 0.05, ***p < 0.01; Standard errors displayed in parentheses; Coefficients compared to the reference category (R) in brackets;^1)^ Need to borrow/spend savings or can’t buy at all things needed.

PCH, personal connections in healthcare; IPH, informal payments in healthcare; HU, healthcare users.

*Source*: author`s calculations based on data from 2^nd^ (2021) Global Corruption Barometer (GCB)—EU [1].

Having explained the importance of considering the selection issue, the analysis now turns to the analysis of the results of the scenarios related to the informal practices used for accessing public healthcare services.

Starting with those who used both personal connections and informal payments (Scenario A), the finding is that women are more likely than men to use these type of practices as well as younger people. Similarly, those more educated make use of informal practices more than those less educated. Meanwhile, those retired, those not working and homemakers are more likely to use informal practices for accessing public healthcare services than those employed. Those with financial difficulties are more likely to use informal practices and so too are those living in towns compared with those living rural areas or villages.

As the Hypothesis 1 asserted, the results show that indeed, those with a medium or high asymmetry level are more likely to use informal practices to access public healthcare services than those with a low asymmetry level, confirming this hypothesis. Similarly, Hypothesis 2 is confirmed, those with a higher level of trust in public authorities are less likely to employ such informal practices than those with a higher level of trust in public authorities (Scenario A, Model 1). Turning to those who use the two informal practices for accessing public healthcare services on a regular basis (i.e., they do so frequently and not only once or twice as an exceptional event), broadly the same findings are identified, except the fact that gender and occupation do not have a significant association anymore. However, the role of the institutional asymmetry and of trust in public authorities remains unchanged, confirming the Hypothesis 1 and Hypothesis 2 in Scenario B (Model 2).

Moving to those who use personal connections but not informal payments (Model 3, Scenario C), the results show that youngsters are more likely to use their connections than other older groups and so too are the students compared with those in employment and those with financial difficulties compared with those affording to buy what they want. This indicate that those with limited financial possibilities as students or those struggling financially use rather their connections alone and do not afford informal payments in additions. Meanwhile, those living in large towns are less likely to use their connections to access public healthcare services. When investigating the link between the prevalence of using connections to access public healthcare services and the level of asymmetry between formal and informal institutions, the previous results remain valid, confirming Hypothesis 1 and 2 (Scenario C, Model 3). Moving to those who use this practice more often, the results show that women are more likely to use their connections compared with men. Similarly, youngsters as well as those with financial difficulties are more likely to use their connections. Meanwhile, those more educated are less likely to use their connections than those less educated. Turning to the role of institutional asymmetry and the level of public trust, the association is again confirmed, validating the Hypothesis 1 and Hypothesis 2 in Scenario D (Model 4). Due to the low number of cases and consequently low reliability of the regression results, no multivariate analysis could be performed for Scenario E and F.

In sum, the results of the regression analysis show that regardless of the type of informal practice analyzed, there is a strong relationship between the prevalence of these practices and the asymmetry between the formal and informal institutions and the trust in government. As Table 5 displays, the results are robust and these relationships remain unchanged when using alternative methods to analyze the data namely, probit regression with sample selection for imputed data and multilevel mixed-effects probit regression.

**Table 5:** Robustness checks (Global Corruption Barometer—European Union, Europe, 2021).

	Probit regression (with sample selection)				Multilevel mixed-effects probit regression			
	Imputed missing data							
	Scenario A	Scenario B	Scenario C	Scenario D	Scenario A	Scenario B	Scenario C	Scenario D
Control Variables								
Socio-demographic variables	Yes	Yes	Yes	Yes	Yes	Yes	Yes	Yes
Institutional asymmetry								
Asymmetry formal-informal institutions (R: Low asymmetry level)								
Medium asymmetry level	0.167*** (0.047)	0.196*** (0.063)	0.042 (0.034)	0.026 (0.039)	0.088 (0.058)	0.119 (0.077)	0.066* (0.036)	0.059 (0.045)
High asymmetry level	0.344*** (0.032)	0.368*** (0.043)	0.119*** (0.024)	0.098*** (0.028)	0.263*** (0.041)	0.285*** (0.053)	0.139*** (0.026)	0.155*** (0.033)
Trust in Public Authorities Index	−0.990*** (0.054)	−1.076*** (0.075)	−0.456*** (0.036)	−0.514*** (0.042)	−0.922*** (0.071)	−1.014*** (0.095)	−0.507*** (0.038)	−0.545*** (0.048)
Selection equation with socio-demographic variables	Yes	Yes	Yes	Yes				
Observations	33,893	33,109	39,254	36,189	18,015	17,262	23,144	20,189
Imputations (multivariate)	10	10	10	10				
Variance at country level (ICC) (%)				7	6	3	6	

*Notes*: Significant at *p < 0.1, **p < 0.05, ***p < 0.01; Coefficients compared to the reference category (R) in brackets; Standard errors displayed in parentheses.

*Source*: author`s calculations based on data from 2^nd^ (2021) Global Corruption Barometer (GCB)—EU [1].

The predicted probability to engage in informal practices for a “representative” patient in Europe further reinforce these findings. The “representative” patient in Europe has been obtain by using the mean and the mode of the control variables used in the analysis. As Supplementary Figure S1 displays, regardless whether we analyze the informal practices together or if we analyze solely the use of connections for accessing the public healthcare services, the predicted probability to make use of these practices increase with a high asymmetry between formal and informal institutions and a low trust in public authorities.

## Discussion

This paper has explored an aspect of corruption in the health services sector [42–47], namely the informal practices of making informal payments and personal connections to gain preferential access to healthcare services during the COVID-19 pandemic. Metaphorically, this corruption can itself be seen as an ignored pandemic in the present-day health services sector [11,13,48–50].

This paper has revealed the magnitude of the use of informal payments and personal connections to gain access to health services during the COVID-19 pandemic in the European Union. It has also revealed, based on institutional theory, an explanation for these informal practices. It has been shown that when there is asymmetry between formal and informal institutions, informal practices are more prevalent. Institutional asymmetry is greater, meanwhile, when there is a greater lack of trust in public institutions.

To tackle informal payments therefore, it will be necessary to address this institutional asymmetry. This requires changes in on the one hand, the personal norms that constitute the informal institutions [14,51–53] but also the formal institutions [14,54,55]. To change the personal norms that view informal practices as acceptable (i.e., the informal institutions), three policy initiatives are required. Firstly, social marketing campaigns are required targeting the groups identified above with high levels of institutional asymmetry. These need to inform patients of the costs and risks of engaging in informal practices. Secondly, normative appeals to medical staff can be used to try to curb the tendency to engage in such practices. And third and finally, education is required to inform staff and patients of the benefits of not engaging in informal practices. Formal institutions must also change. Informal practices are more common in systems where there is low public trust in formal institutions. There is therefore a need to modernise healthcare services to improve trust in these institutions.

Nevertheless, this study has limitations. Due to lack of data, this analysis could not control for the total use of public healthcare services nor to evaluate patients’ level of dependence on informal practices when they access public healthcare services. However, based on previous studies, the frequency of using public healthcare services does not raise confounder issues. Firstly, previous studies investigating the relationship between informal practices and healthcare utilisation frequency revealed inconclusive findings [56–58]. These findings are further reinforced by the fact that informal payments are found to be more prevalent for in-patient services [59] and for specific health services (e.g., gynaecology) [56]. Maternal healthcare involves a large number of antenatal visits and an informal payment is usually made at the time of delivery [60]. This therefore suggest that informal practices are a complex phenomenon, not necessarily related with the healthcare utilisation frequency. Secondly, and considering the strong association between trust and satisfaction, past findings revealed either a positive or negative relationship, either no relationship between satisfaction and the number of patient visits [61]. Regarding the asymmetry formal-informal institutions, previous studies identified various factors that can influence the asymmetry level [62], none of them investigating the frequency of using public services. Future research therefore should further investigate the role of healthcare utilisation frequency when analysing informal practices in healthcare.

Moreover, the empirical representative data used in this study does not allow a more nuanced understanding of the causes of the institutional asymmetry and trust in public authorities. As such, future research might address these limitations and further examine causality issues by using longitudinal data or focus on those patients rarely using informal practices.

In sum, if governments aim to tackle informal practices by patients during a public health crisis in a more effective manner, policy measures to reduce the asymmetry between the formal rules and personal norms by raising trust in public authorities should be sought.
