# Supplementary material for: The Companion Pandemic to COVID-19: The Use of Informal Practices to Access Public Healthcare Services in the European Union
Source: Int J Public Health. 2022 Oct 19;67:1604405. doi: 10.3389/ijph.2022.1604405 (PMC9626517; doi:10.3389/ijph.2022.1604405)
Supplement: Supplementary file 1 [file DataSheet1.doc]

**Supplementary File 1**

**Table S1. Scenarios and variables evaluated (N = 25,774; Global Corruption Barometer – European Union, Europe, 2021)**

| Scenario or variable | Details | Descriptive statistics | |
| --- | --- | --- | --- |
|  |  | % Yes, Mode or Mean | Min and Max |
| Scenario A | Dummy variable for patients using both personal connections and informal payments to access public healthcare services. | Both personal connections and informal payments (5%) | 0/1 |
| Scenario B | Dummy variable for patients using frequently (Few times / Often in the last months) both personal connections and informal payments to access public healthcare services. | Both personal connections and informal payments, frequently (2%) | 0/1 |
| Scenario C | Dummy variable for patients using solely personal connections to access public healthcare services. | Solely personal connections (24%) | 0/1 |
| Scenario D | Dummy variable for patients using frequently (Few times / Often in the last months) solely personal connections to access public healthcare services. | Solely personal connections, frequently (12%) | 0/1 |
| Scenario E | Dummy variable for patients using solely informal payments to access public healthcare services. | Solely informal payments (1.8%) | 0/1 |
| Scenario F | Dummy variable for patients using frequently (Few times / Often in the last months) solely informal payments to access public healthcare services. | Solely informal payments, frequently (0.6%) | 0/1 |
| Country | Categorical variable for 27 European Union countries. | Germany (17%) | 1/27 |
| Region | Categorical variable for the European regions: East-Central Europe, Western Europe, Southern Europe and Nordic Nations. | Western Europe (39%) | 1/4 |
| Gender | Dummy variable for patient`s gender. | Female (57%) | 0/1 |
| Age | The exact age of the patient. | 49 years old | 18/105 |
| Education | Dummy variable for the education of the patient: primary/secondary or tertiary education. | Primary/secondary (66%) | 0/1 |
| Occupation | Categorical variable for the occupation of the patient: employed, not working / homemaker, retired, student. | Employed (57%) | 1/4 |
| Household income | Categorical variable for the household income: enough to buy what wanted, enough to buy what is needed, manage with difficulties, not enough to buy what is needed. | Enough to buy what wanted (42%) | 1/4 |
| Residency area | Categorical variable for patient area of the residence: rural area or village, small or middle-sized town, large town. | Small, middle-sized town (35%) | 1/3 |
| Asymmetry formal-informal institutions | Categorical variable for evaluating the asymmetry: low asymmetry level, medium asymmetry level, high asymmetry level. High asymmetry when personal norms, values are not in accordance with formal rules. | Low asymmetry level (77%) | 1/3 |
| Trust in Public Authorities Index | Constructed index of self-reported trust in the public authorities (trust in national government and local government). High score (1) means high trust in public authorities. | Trust in Public Authorities Index: 0.52 | 0/1 |

*Source*: author`s calculations based on data from 2^nd^ (2021) Global Corruption Barometer (GCB) – EU [1]

**Table S2.** Multiple imputations – missing data (Global Corruption Barometer – European Union, Europe, 2021)

| Variable | Complete | Incomplete | Imputed | Total |
| --- | --- | --- | --- | --- |
| Age | 40663 | 0 | 0 | 40663 |
| Gender | 40653 | 10 | 10 | 40663 |
| Education | 40334 | 329 | 329 | 40663 |
| Occupation | 40400 | 263 | 263 | 40663 |
| Household income | 39992 | 671 | 671 | 40663 |
| Residency area | 40443 | 220 | 220 | 40663 |
| Asymmetry formal-informal institutions | 39453 | 1210 | 1210 | 40663 |
| Trust in Public Authorities Index | 40505 | 158 | 158 | 40663 |

*Source*: author`s calculations based on data from 2^nd^ (2021) Global Corruption Barometer (GCB) – EU [1]
