# Supplementary material for: The Companion Pandemic to COVID-19: The Use of Informal Practices to Access Public Healthcare Services in the European Union
Source: Int J Public Health. 2022 Oct 19;67:1604405. doi: 10.3389/ijph.2022.1604405 (PMC9626517; doi:10.3389/ijph.2022.1604405)
Supplement: Supplementary file 2 [file DataSheet2.doc]

**Supplementary File 2**

| **SCENARIO A** | 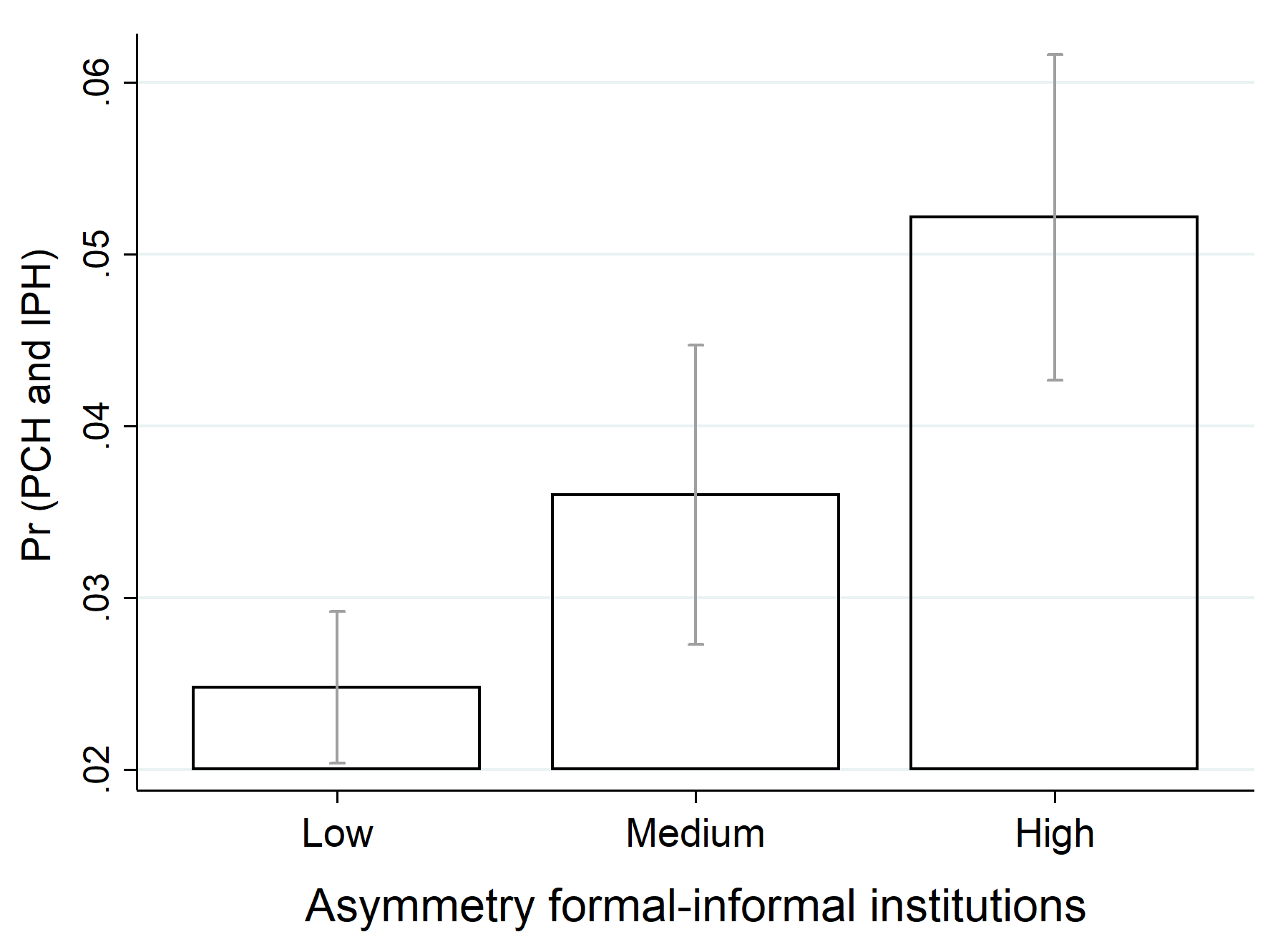 | **A1** | 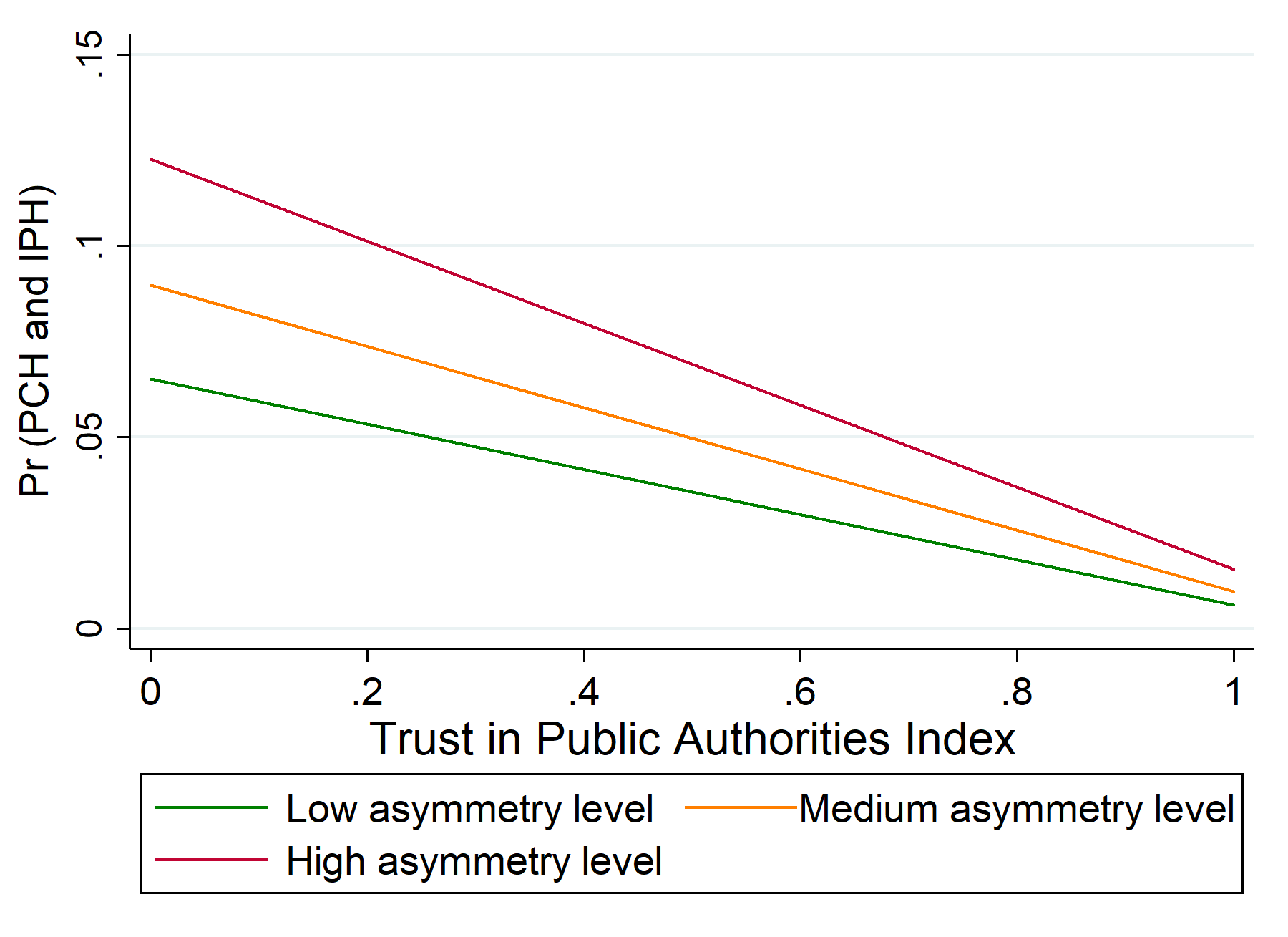 | **A2** |
| --- | --- | --- | --- | --- |
| **SCENARIO B** | 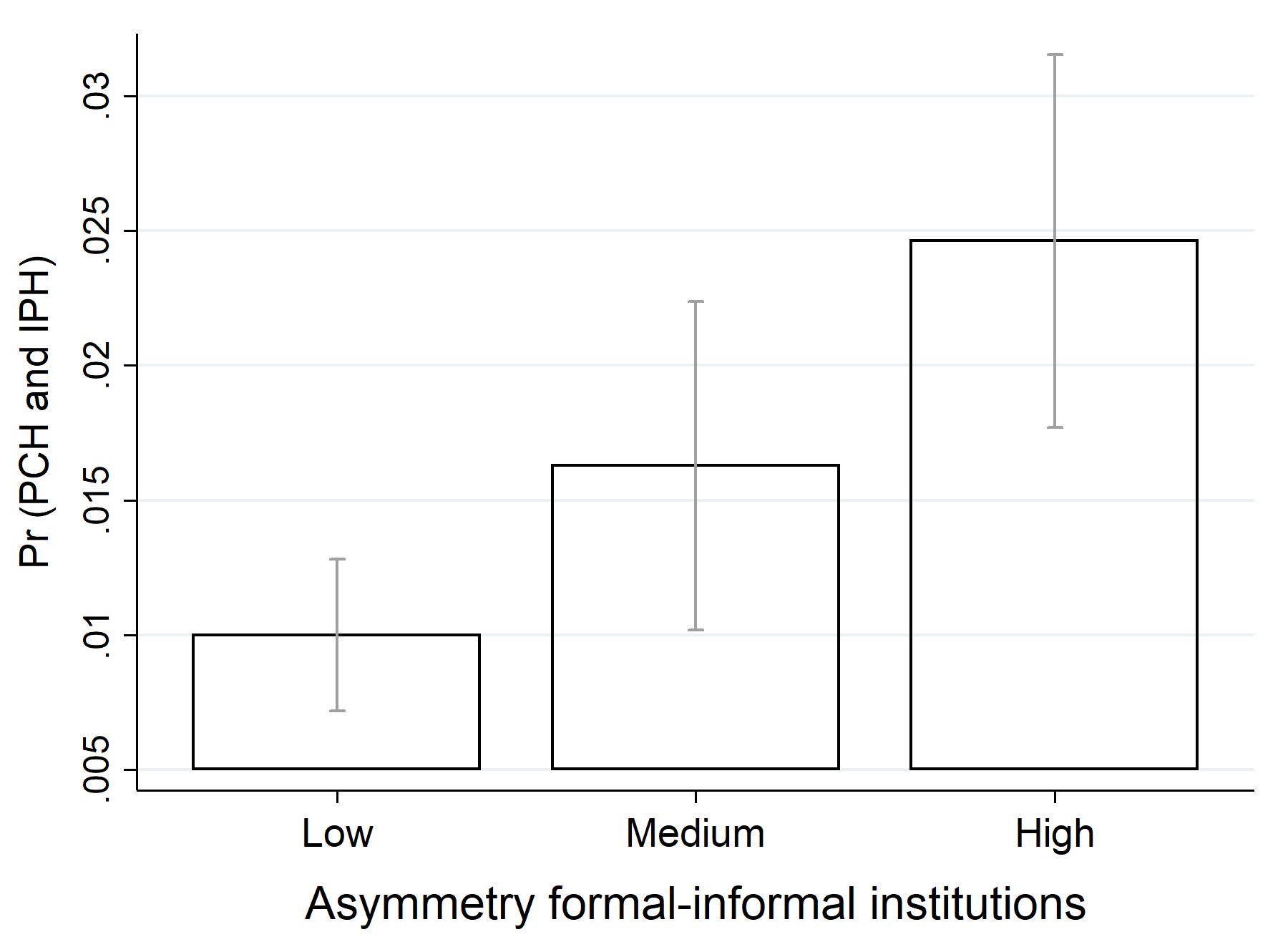 | **B1** | 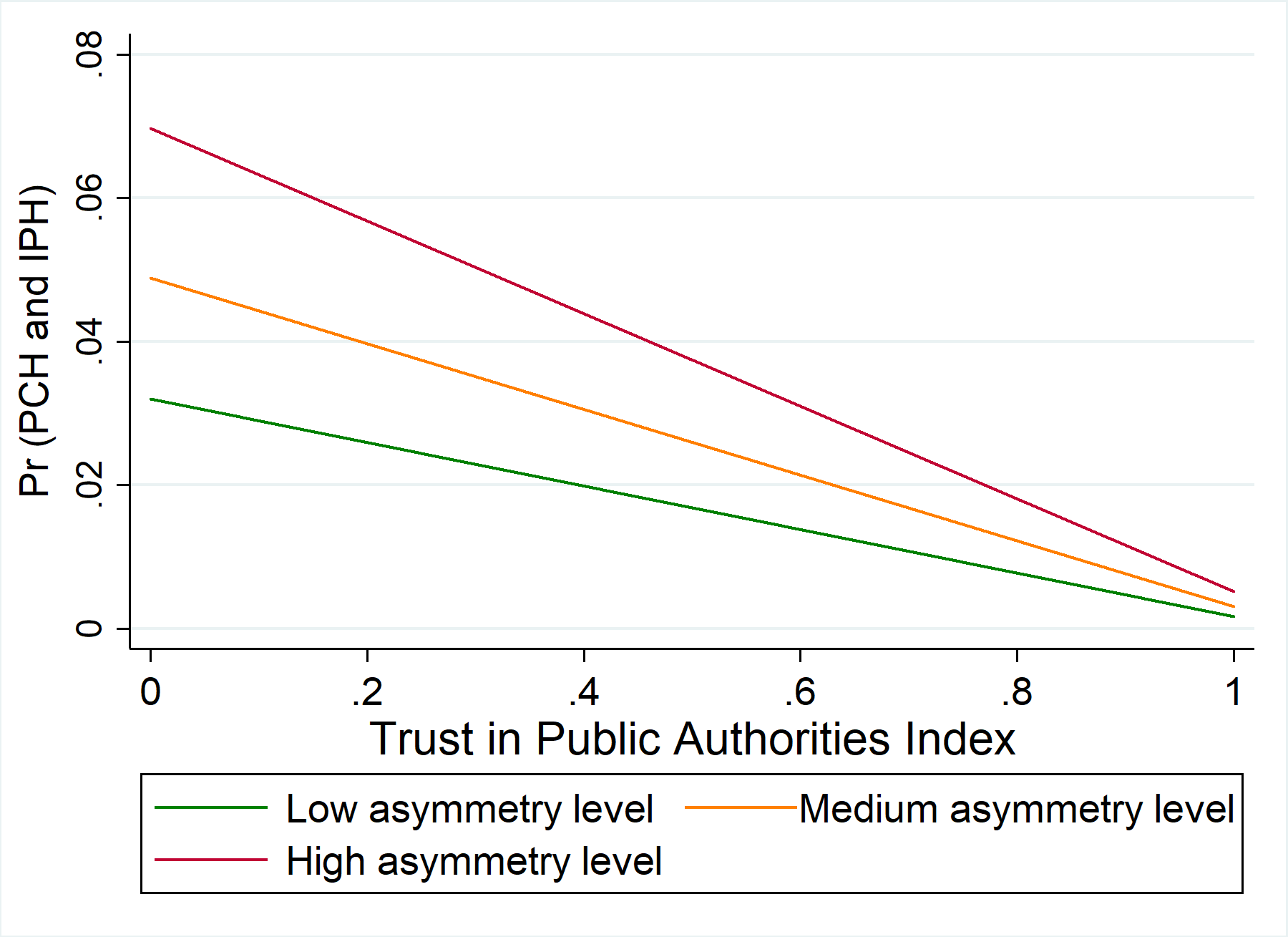 | **B2** |
| **SCENARIO C** | 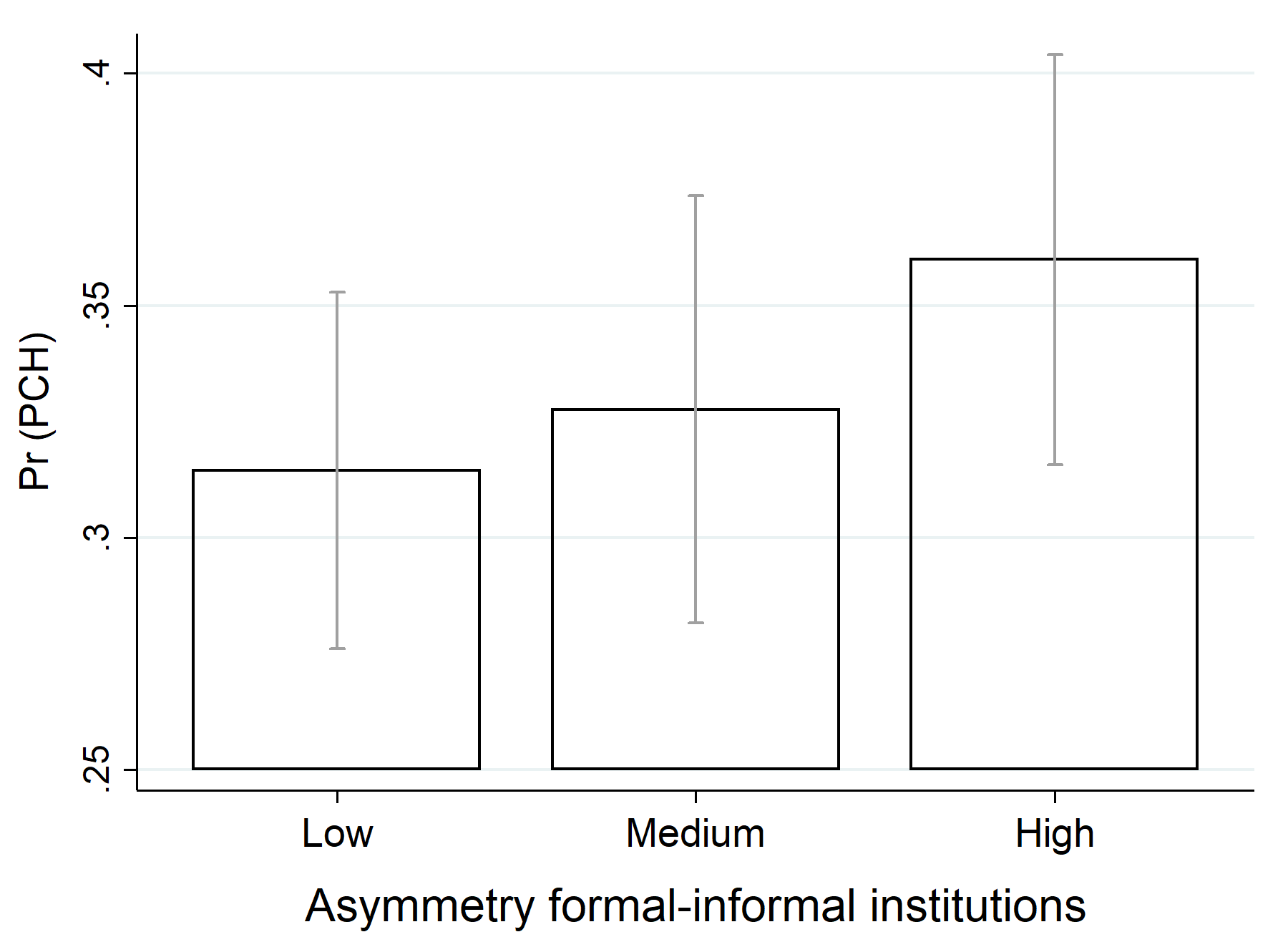 | **C1** | 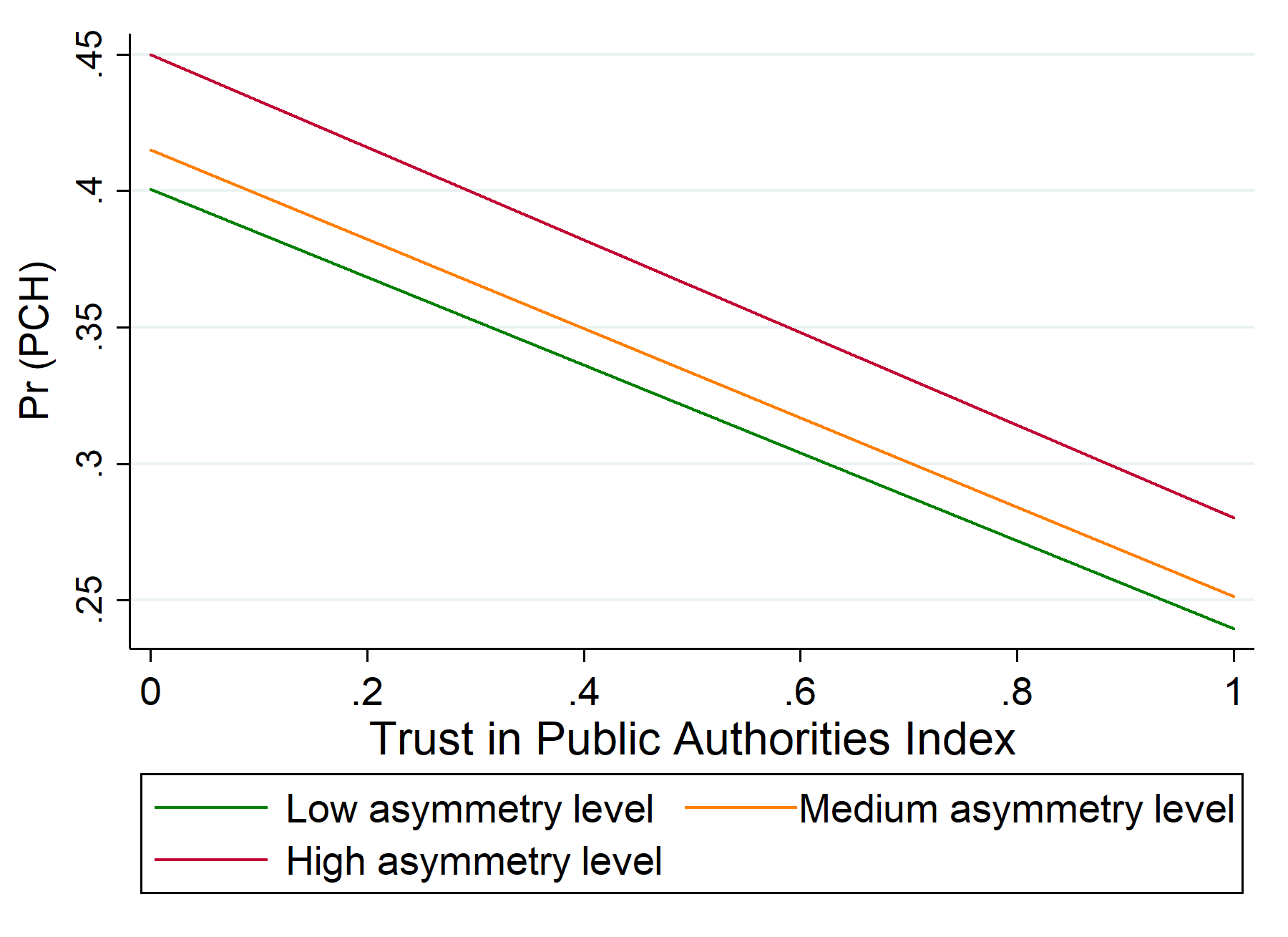 | **C2** |
| **SCENARIO D** | 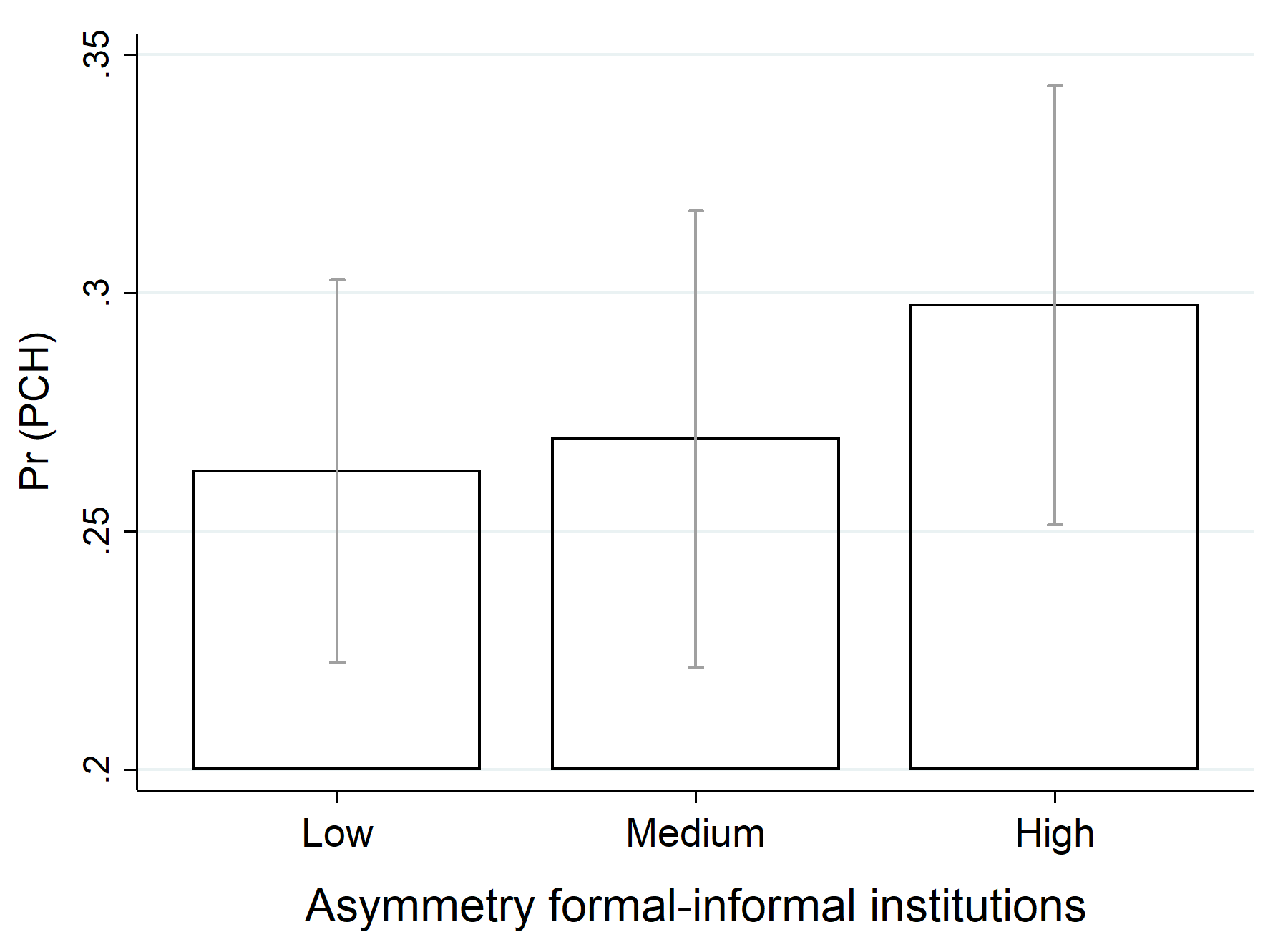 | **D1** | 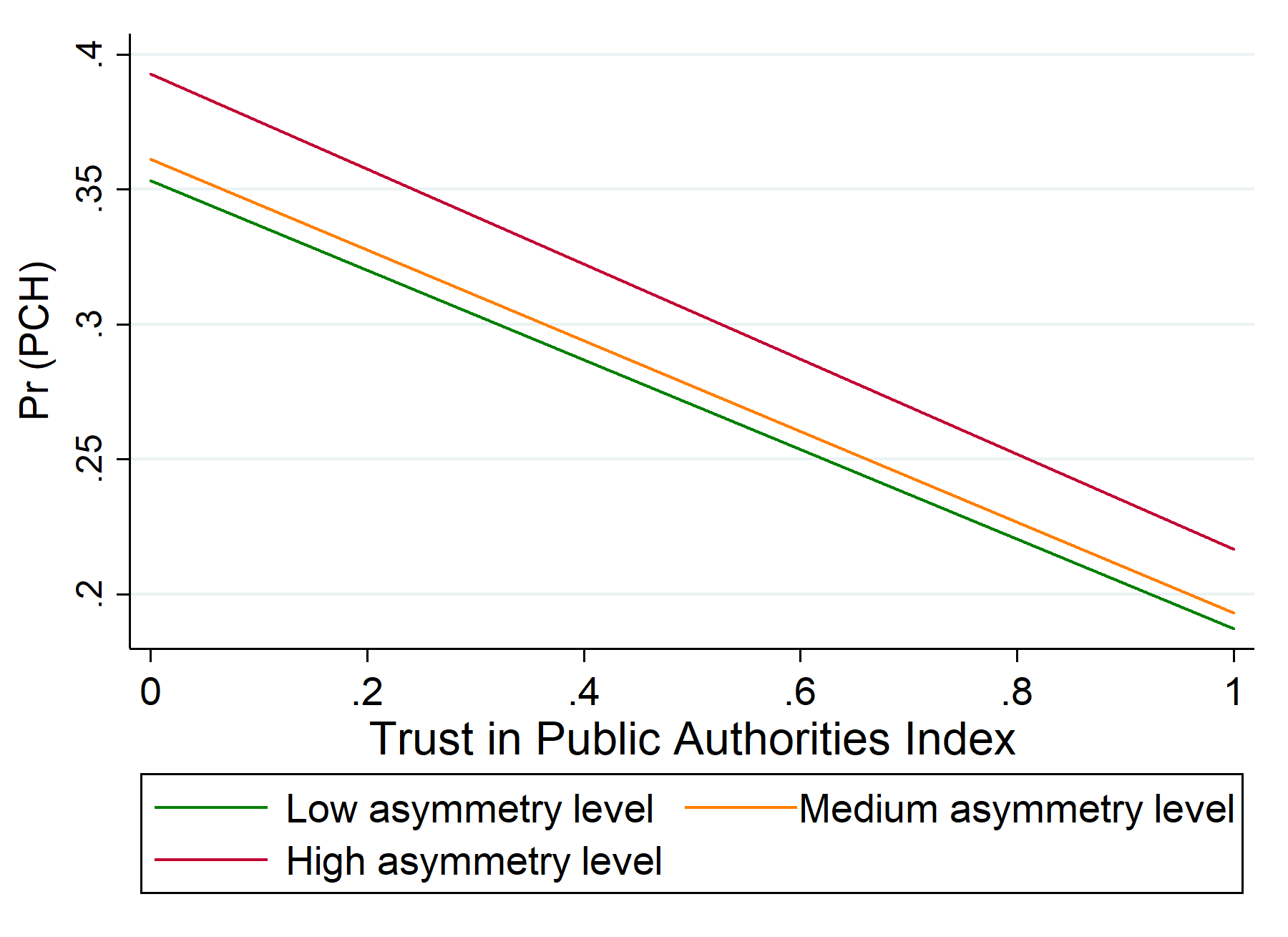 | **D2** |

**Figure S1.** Predicted probability to engage in informal practices in healthcare by a “representative” patient in Europe, by asymmetry formal-informal institutions and trust in public authorities (Global Corruption Barometer – European Union, Europe, 2021)

*Notes*: PCH = personal connections in healthcare; IPH = informal payments in healthcare

*Source*: author`s calculations based on data from 2^nd^ (2021) Global Corruption Barometer (GCB) – EU [1]
